# Supplementary material for: Side-group chemical gating via reversible optical and electric control in a single molecule transistor
Source: Nat Commun. 2019 Mar 29;10:1450. doi: 10.1038/s41467-019-09120-1 (PMC6440973; doi:10.1038/s41467-019-09120-1)
Supplement: Supplementary file 1 — Supplementary Information [file 41467_2019_9120_MOESM1_ESM.pdf]

Supplementary Information

Side–Group Chemical Gating via Reversible Optical and Electric Control in a Single Molecule Transistor

Linan Meng,[1,2,3†] Na Xin,[2†] Chen Hu,[4†] Jinying Wang,[2] Bo Gui,[5] Junjie Shi,[6] Cheng Wang,[5] Cheng Shen,[1] Guangyu Zhang,[1] Hong Guo,[4*] Sheng Meng,[1,3*] Xuefeng Guo[2,7*]

[1]Institute of Physics, Chinese Academy of Sciences, Beijing 100190, P. R. China.

[2]Beijing National Laboratory for Molecular Sciences, State Key Laboratory for Structural Chemistry of Unstable and Stable Species, College of Chemistry and Molecular Engineering, Peking University, Beijing 100871, P. R. China.

[3]University of Chinese Academy of Sciences, Beijing 100049, P. R. China.

[4]Center for the Physics of Materials and Department of Physics, McGill University, Montreal, Quebec H3A 2T8, Canada.

[5]Key Laboratory of Biomedical Polymers of Ministry of Education, College of Chemistry and Molecular Sciences, Wuhan University, Wuhan 430072, P. R. China.

[6]School of Chemistry & Chemical Engineering, Shandong University, Jinan 250100, Shandong, P. R. China.

[7]Department of Materials Science and Engineering, College of Engineering, Peking University, Beijing 100871, P. R. China.

[†]These authors contributed equally to this work.

*Correspondence author. E–mail: guoxf@pku.edu.cn (X.G.); smeng@iphy.ac.cn (S.M.); hong.guo@mcgill.ca (H.G.).

Table of contents

1. **Supplementary Note 1 ……………………………………………………...S3**
2. **Supplementary Figures 1–14…………………………………………………S4**
3. **Supplementary Tables 1–4……………………………………………………S15**
4. **Supplementary References………………………………….……………...S18**

1. Supplementary Note 1

Analysis of single-molecule connection: As presented elsewhere[1–3], the number of junctions that contribute to charge transport can be carried out by calculating the probability of the connected devices with n–rejoined junctions (G_n) with the binomial distribution and the optimized connection yields:

$$G_\mathrm{n} = \frac{m!}{n!\,(m-n)!} p^n (1-p)^{m-n} \qquad n = 0, 1, 2 \cdots, m$$

Supplementary Equation 1

where m is the number of graphene point contact pairs (210 in the current case) and p is the probability of successful connection for a random junction. Therefore, the possibility of connected junction γ_c can be attained:

$$\gamma_c = 1 - G_0 = 1 - \frac{m!}{0!\,(m-0)!} p^0 (1-p)^m = 1 - (1-p)^m$$

Supplementary Equation 2

where G_0 is the probability of devices without any connected junctions. In our experiments, ~5% junctions show successful connection of molecules and the corresponding possibility of successful connection for each contact pairs (p) is $0.05/m$. Then, the ratio of single–junction devices to the overall reconnected devices is ~97.5%. These results from calculations suggest that, in most cases, charge transport in these devices arises mainly in a single–molecule junction.

2. Supplementary Figures 1–14

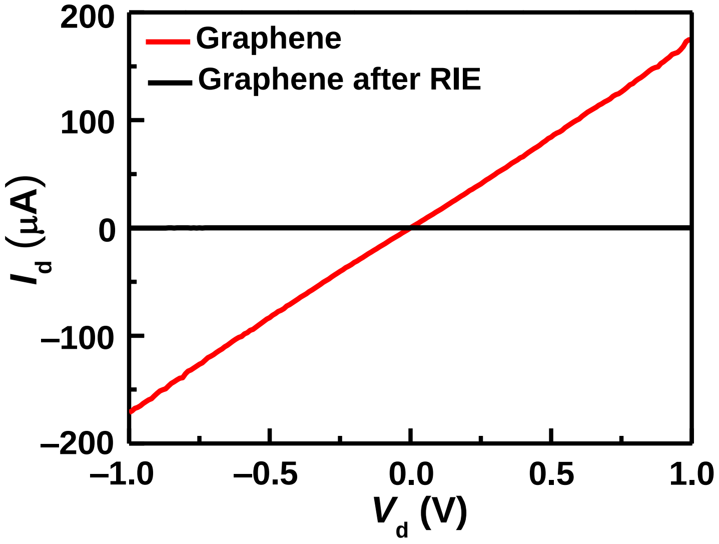

Supplementary Figure 1 | *I*–*V* curves before (red) and after (black) oxygen plasma etching. The gate voltage is 0 V.

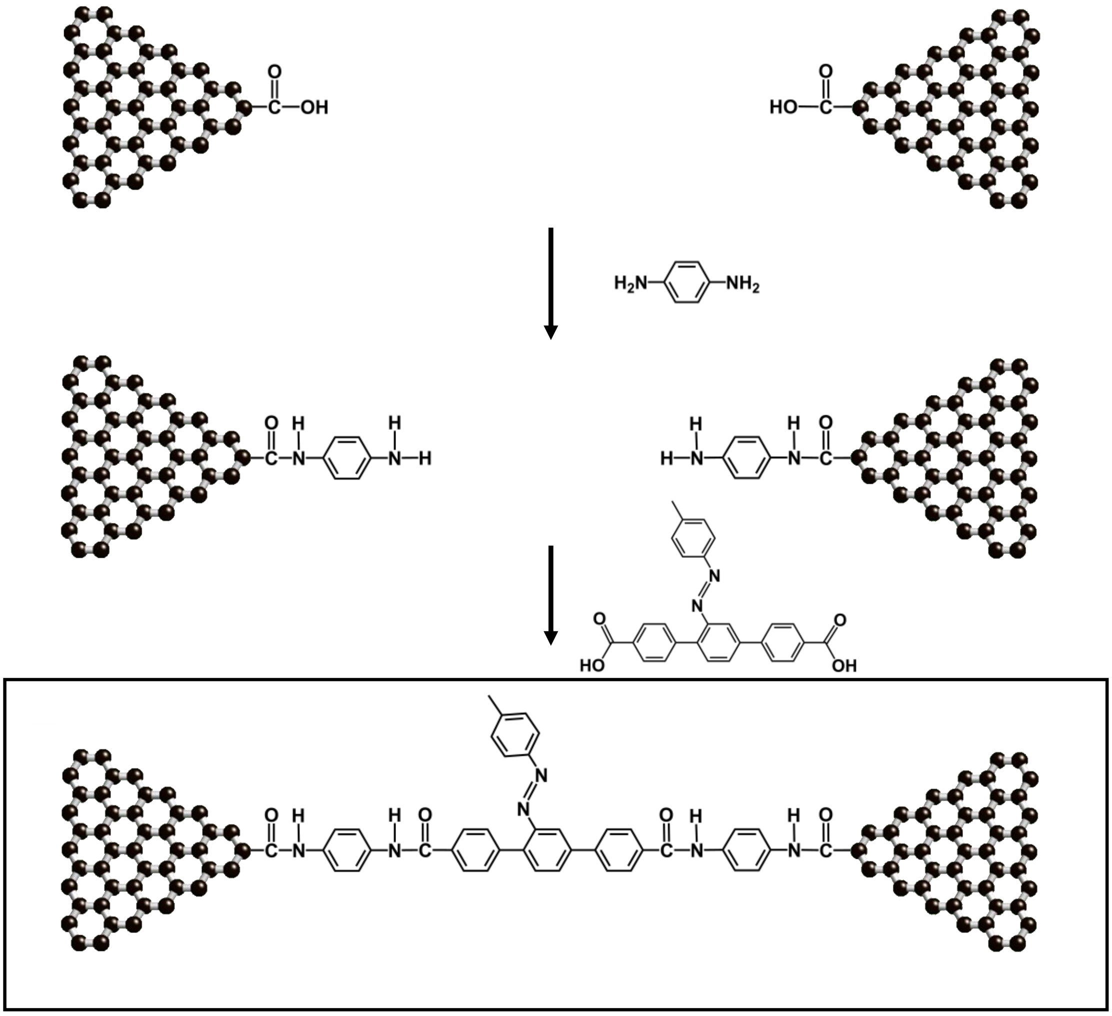

Supplementary Figure 2 | The procedure to connect individual TTDAs with graphene electrodes in two steps.

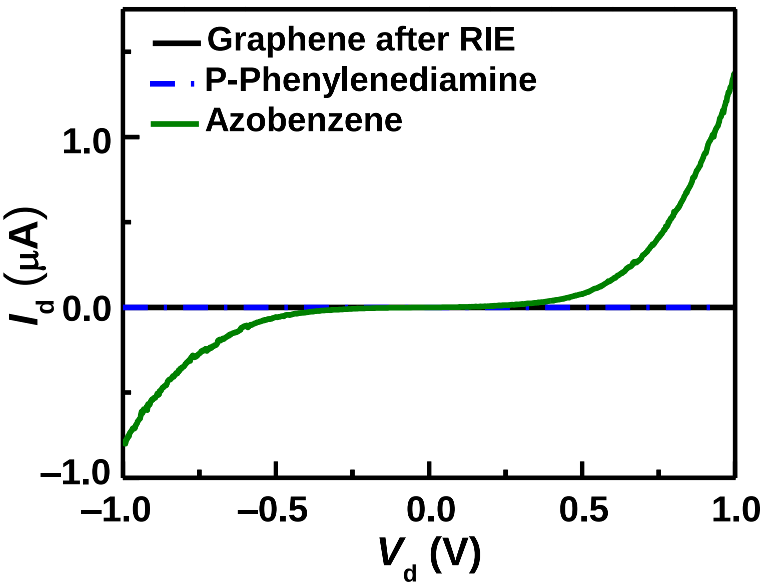

Supplementary Figure 3 | *I–V* curves of a device at different stages: nanogapped graphene (black solid line), p-phenylenediamine-connected graphene (blue dash line) and TTDA-connected graphene (green solid line). The gate voltage is 0 V.

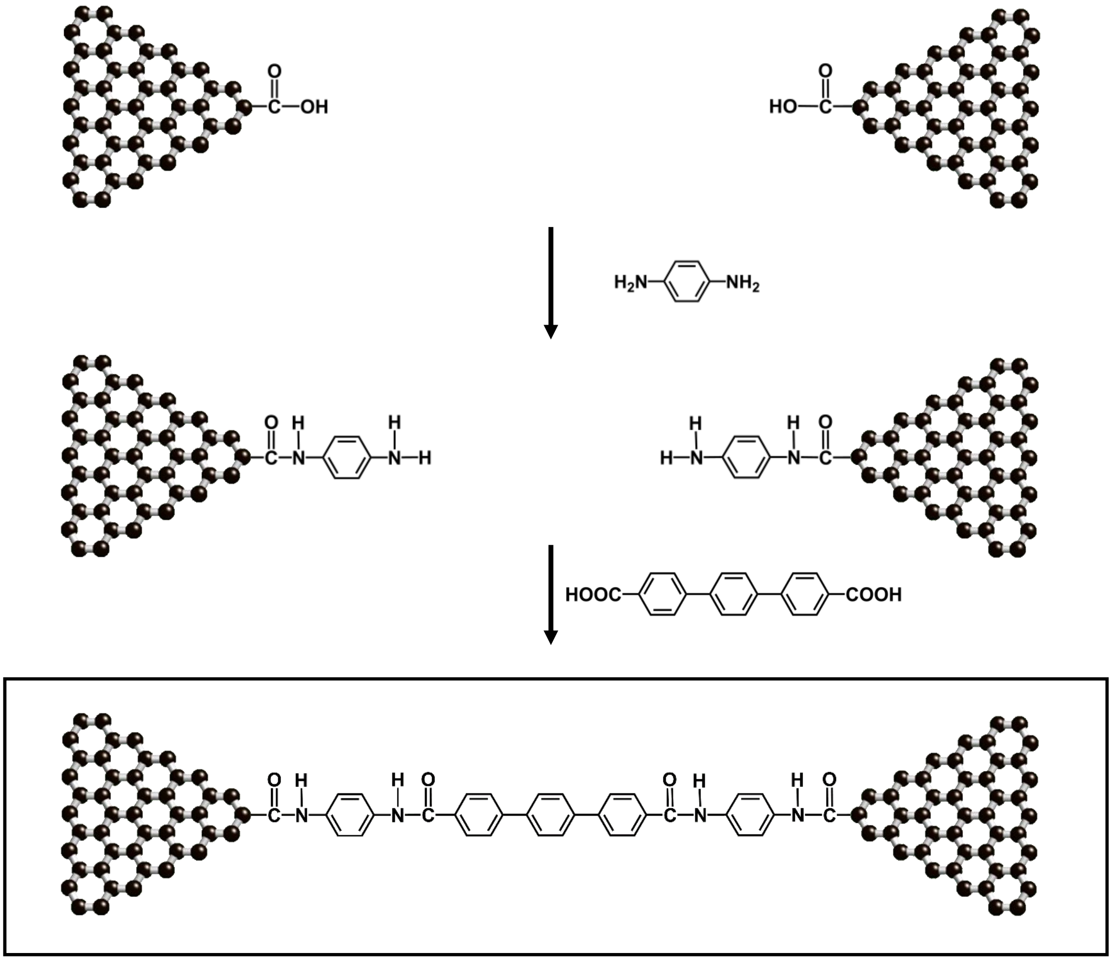

Supplementary Figure 4 | The procedure to connect individual control terphenyl molecules with graphene electrodes in two steps.

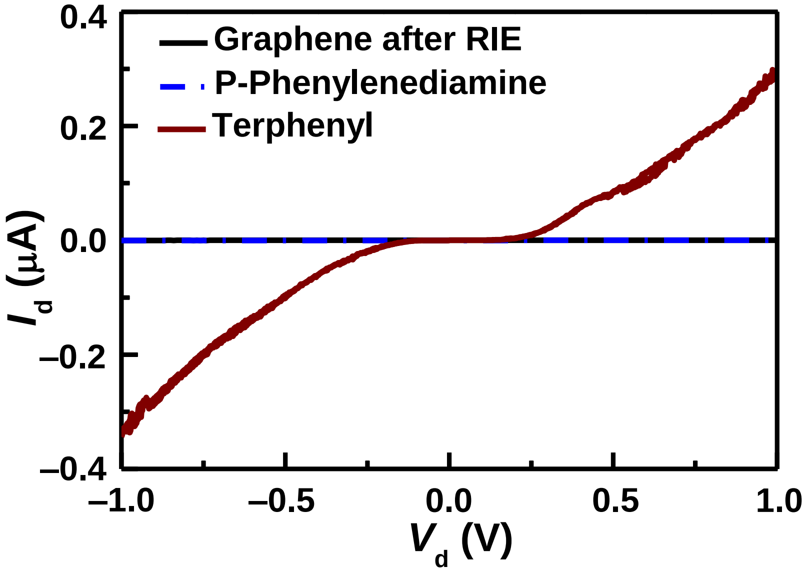

Supplementary Figure 5 | *I–V* curves of a control device at different stages: nanogapped graphene (black solid line), p-phenylenediamine-connected graphene (blue dash line) and control molecule-connected graphene (red solid line). The gate voltage is 0 V.

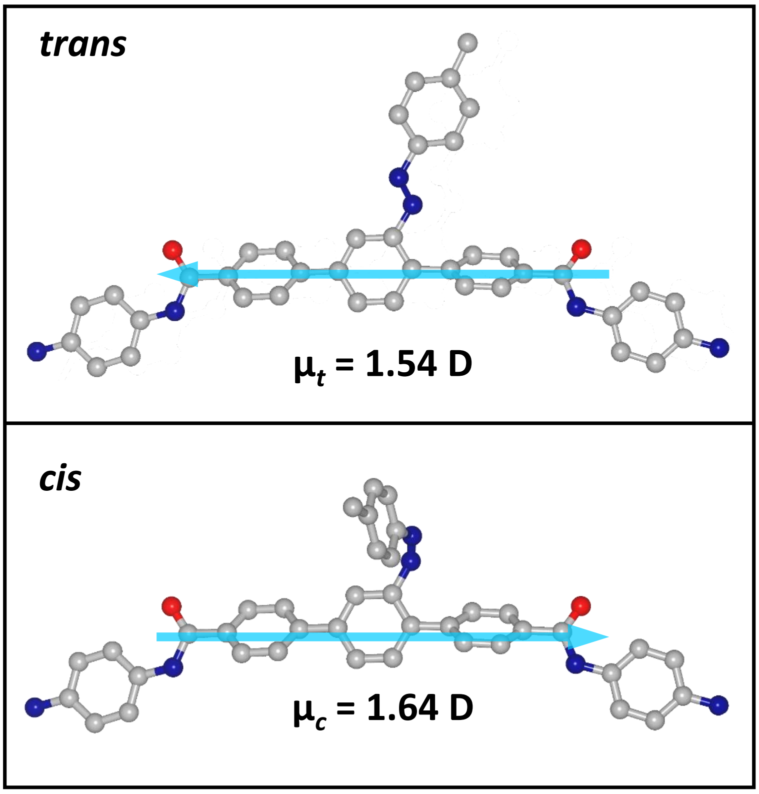

Supplementary Figure 6 | Dipole properties of a phenyl-TTDA-phenyl molecule with *trans* and *cis* conformations.

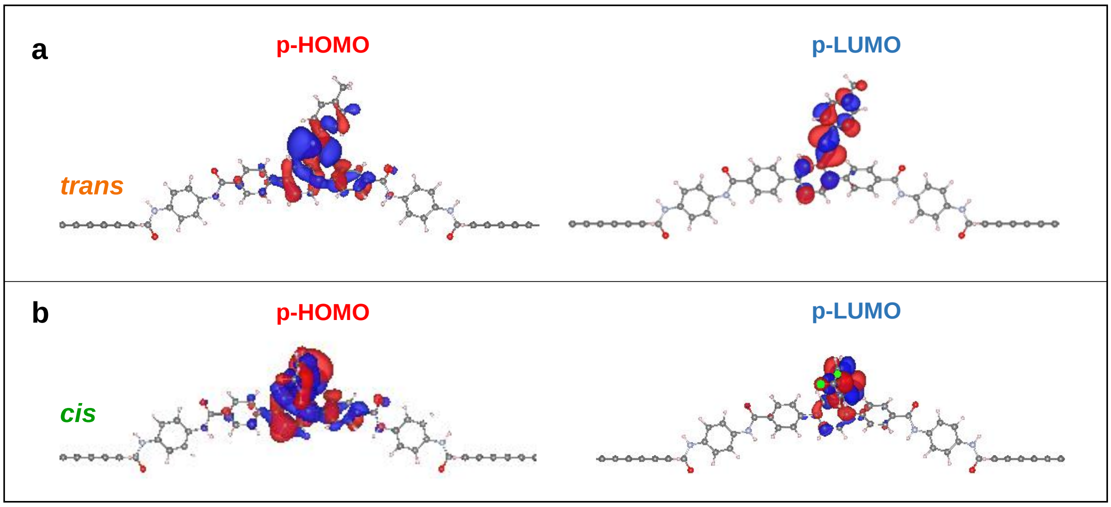

Supplementary Figure 7 | Density of scattering state distribution for p-HOMO and p-LUMO of a TTDA single-molecule junction with *trans* and *cis* forms at the zero bias voltage.

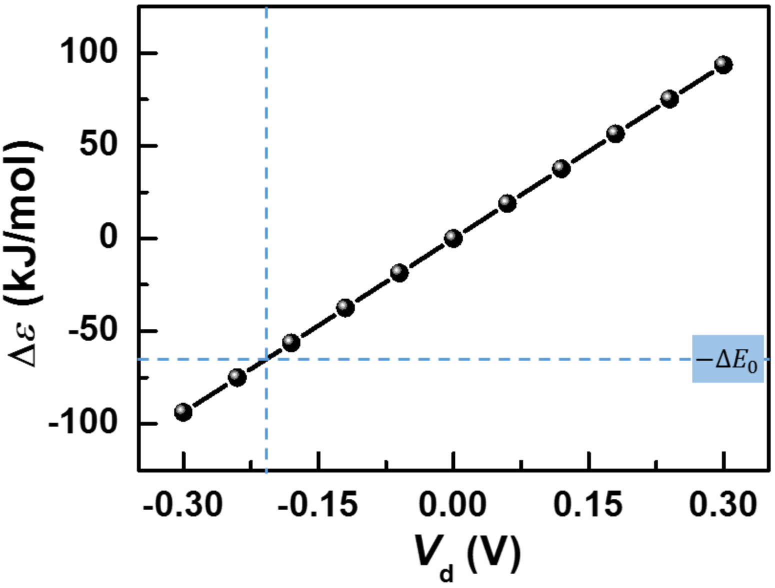

Supplementary Figure 8 | The calculated bias voltage-dependent electrical potential energy differences between *trans* and *cis* forms of a phenyl-TTDA-phenyl molecule. The gate voltage is 0 V.

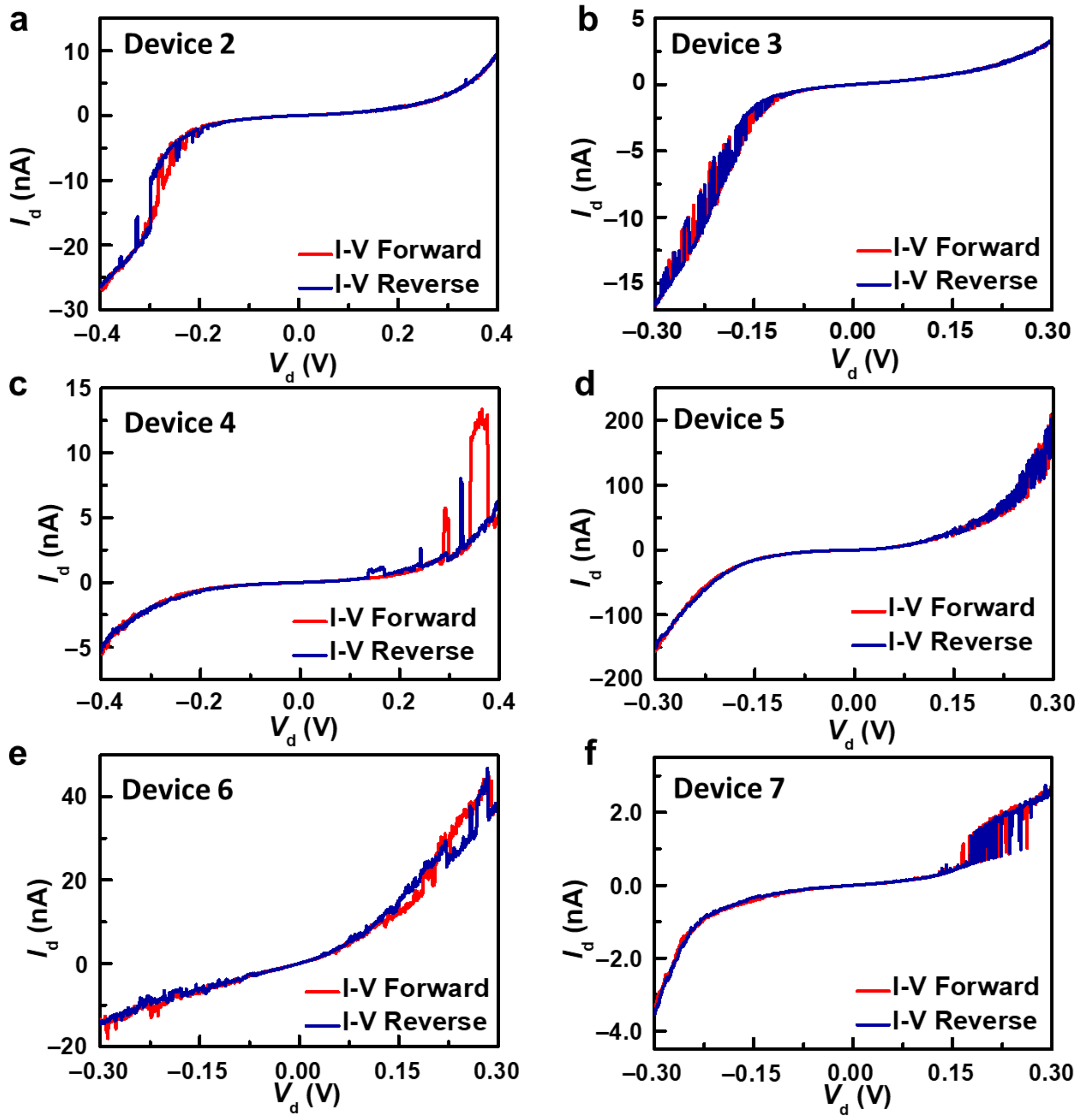

Supplementary Figure 9 | Another six working devices based on TTDA, showing the isomerisation of azobenzene under negative or positive bias voltages due to the orientation alignment of the azobenzene sidegroup relative to graphene source and drain electrodes in comparison with what is presented in Figs. 5d and 5e. The gate voltage is 0 V.

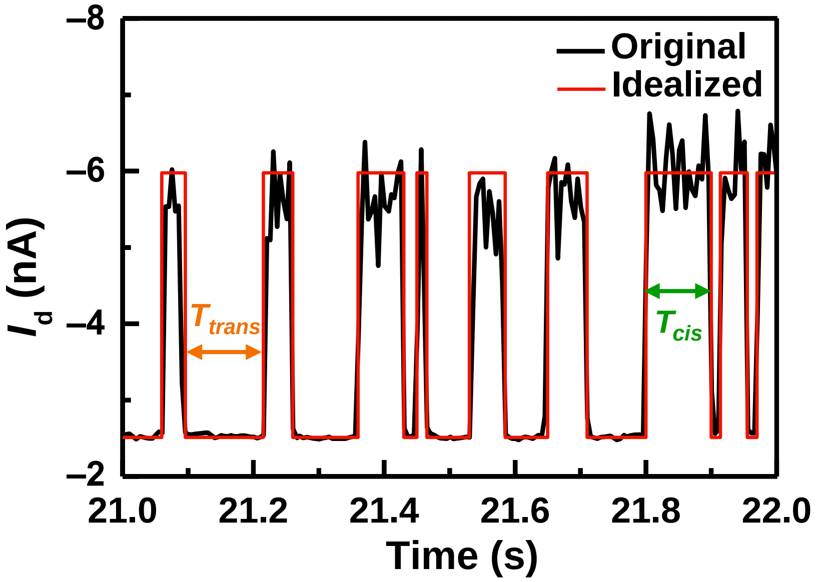

Supplementary Figure 10 | Kinetic analysis. *I*–*t* curve (black) of a GMG-SMJ at V_d = –0.15 V and 160 K and the idealised fit (red) obtained from a segmental *k*-means method based on the hidden Markov model analysis by using a QUB software. The gate voltage is 0 V.

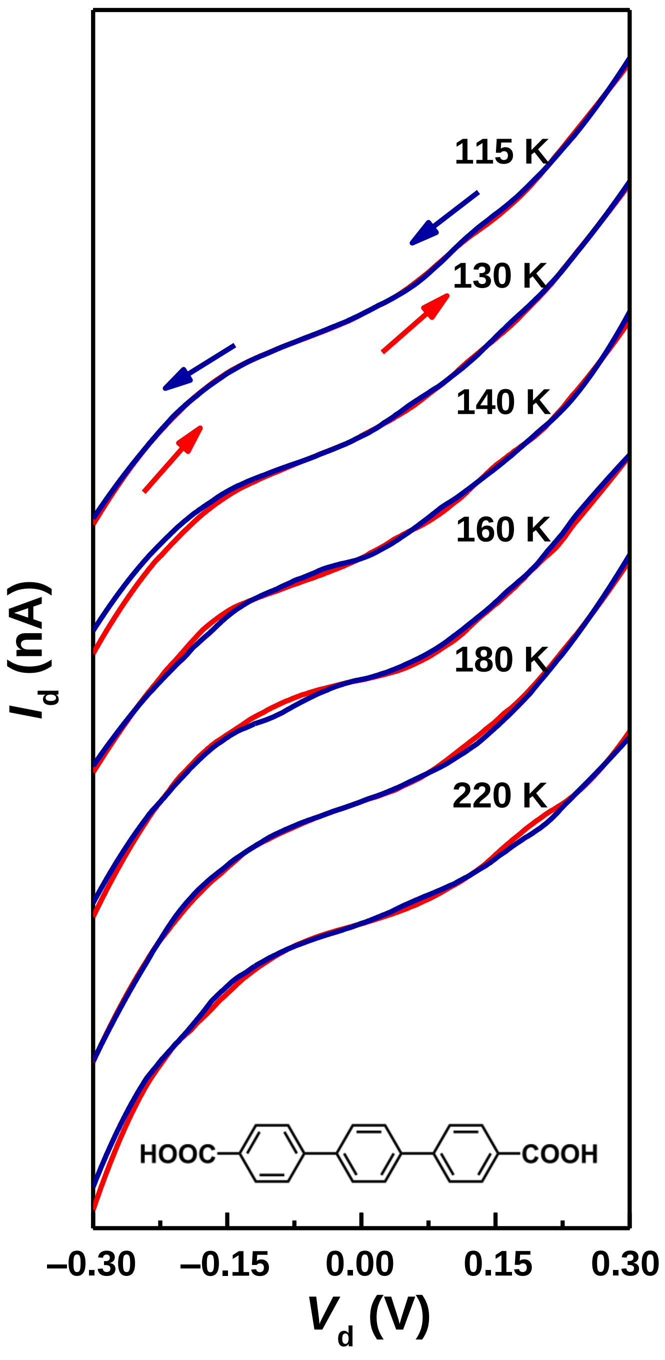

Supplementary Figure 11 | *I*–*V* characteristics for a control terphenyl single–molecule junction at different temperatures. The gate voltage is 0 V.

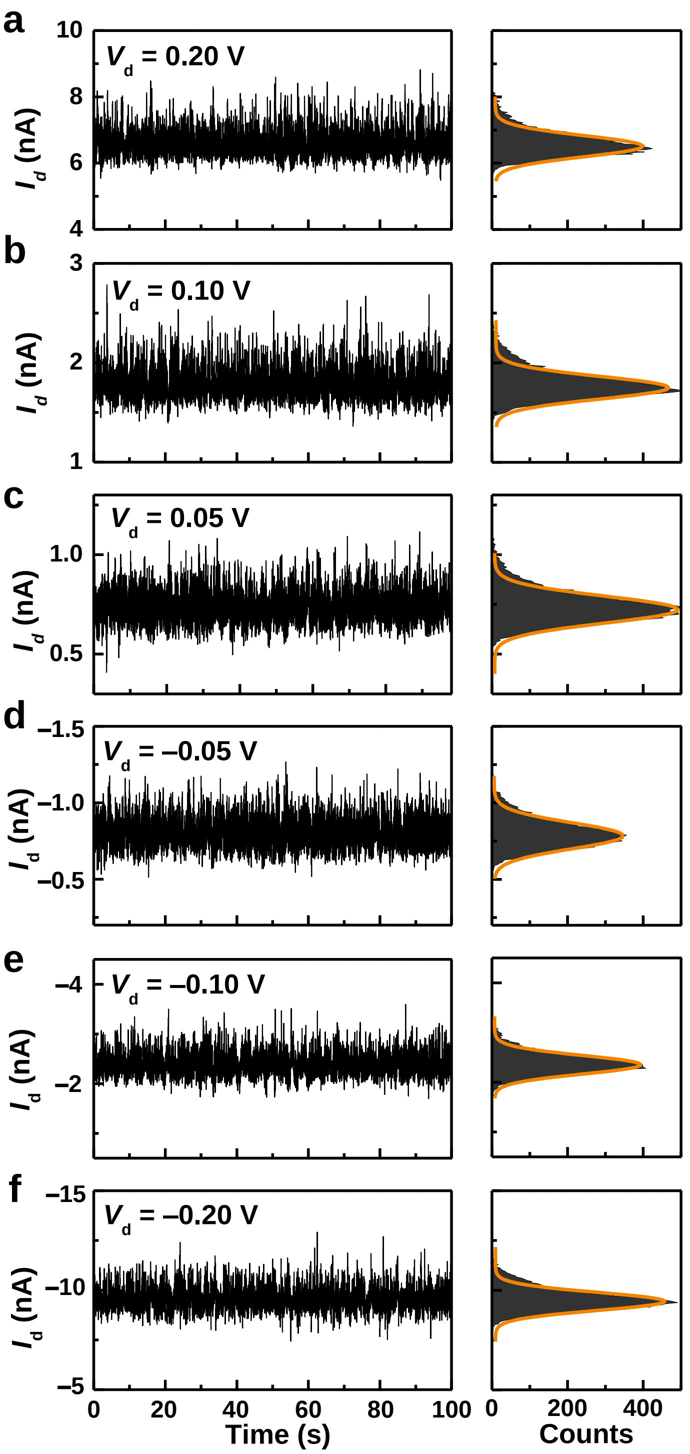

Supplementary Figure 12 | *I-t* characteristics of control terphenyl molecular junctions at different bias voltages. The time-trace currents were carried out at 160 K. Bias voltages were set as 0.20 V, 0.10 V, 0.05 V, –0.05 V, –0.10 V, –0.20 V from a to f. The right panels are the corresponding current histograms.

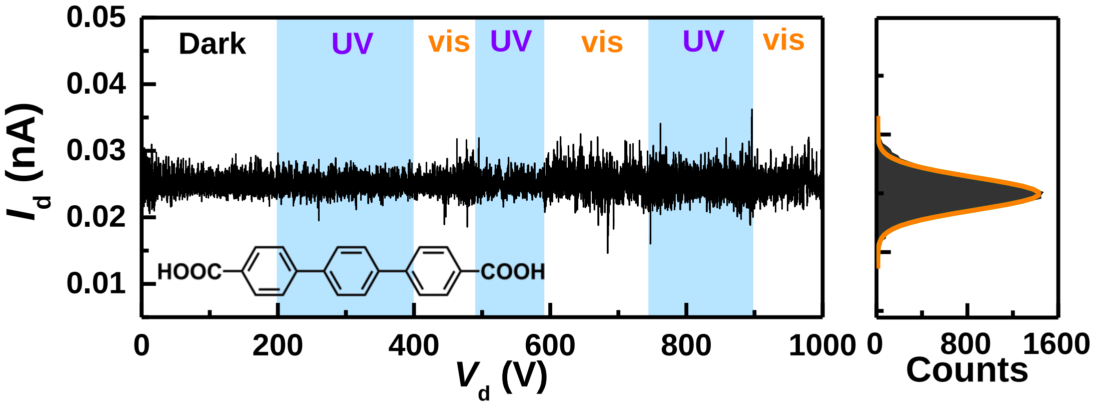

Supplementary Figure 13 | *I–t* characteristics of control terphenyl molecular junctions under sequent illumination. A small positive voltage of 0.01 V was adopted to measure the real–time currents. No switching was observed for the control molecule without the substituted azobenzene sidegroup.

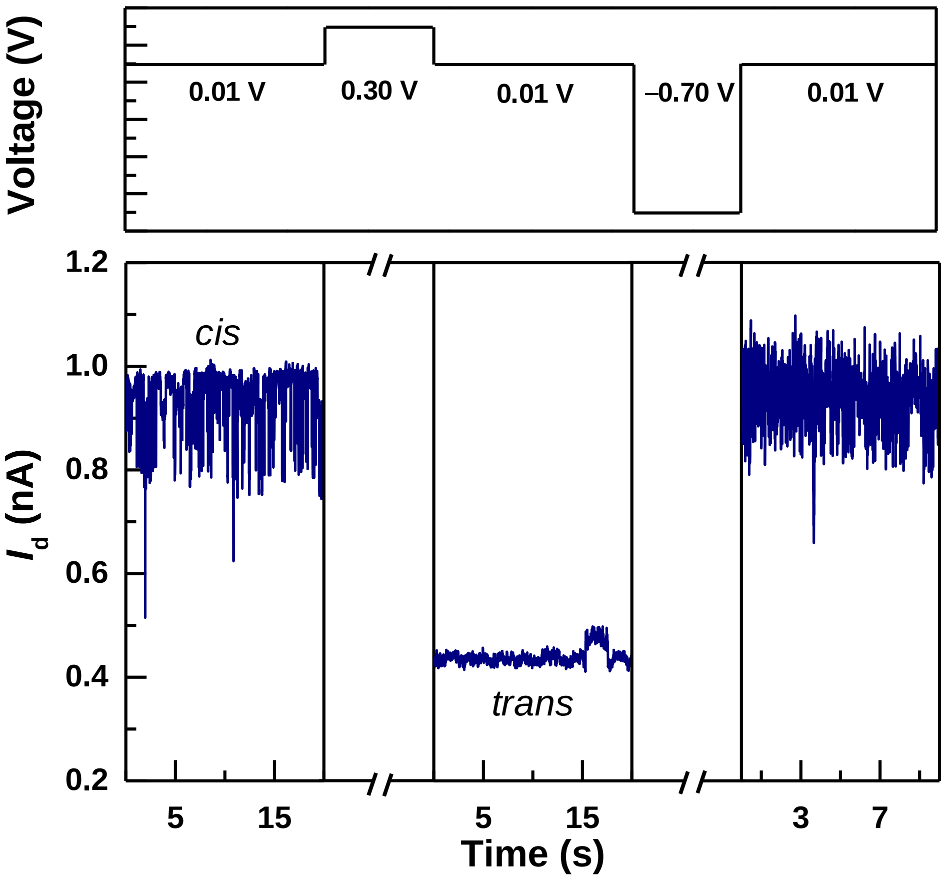

Supplementary Figure 14 | Memory effect based on voltage-induced isomerisation in another device. A small positive voltage of 0.01 V was used to supervise the conductance state and large voltages was used to trigger the isomerisation.

3. Supplementary Tables 1–4

Supplementary Table 1 | Current statistics for both *trans* and *cis* isomers and the switching ratio. NA, not applicable.

V_d (V)	I_{trans} (nA)	I_{cis} (nA)	I_{cis}/I_{trans}
–0.05	–0.59	–1.45	2.46
–0.10	–1.40	–3.41	2.44
–0.15	–2.53	–5.89	2.33
–0.20	–4.39	–8.78	2.00
–0.30	NA	–18.86	NA

Supplementary Table 2 | Kinetic parameters of stochastic switching at different bias voltages. NA, not applicable.

Bias Voltage	Occurrence ratio (%)		$<\tau_{Low}>$ (ms)	$<\tau_{High}>$ (ms)
	low state	high state		
–0.05 V	95%	5%	842.37	24.13
–0.10 V	70%	30%	142.39	47.31
–0.15 V	20%	80%	52.90	74.06
–0.20 V	12%	88%	8.50	95.11
–0.30 V	0%	100%	NA	NA

Supplementary Table 3 | Summary of the resistance values of rejoined junctions based on TTDA. Devices 1-7 showed the electric field-induced switching while Devices 8-9 failed.

Device number	Current (nA)[a]	Molecular resistance (MΩ)[b]	Molecular conductance ($10^{-4} \times G_0$)
1	62.85	7.96	16.22
2	32.97	15.17	8.51
3	29.58	16.90	7.63
4	25.25	19.80	6.52
5	592.30	0.84	152.85
6	83.86	5.96	21.64
7	12.98	38.53	3.35
8	109.43	4.57	28.24
9	140.52	3.56	36.26

[a]The current values are obtained by averaging the data from *I-V* curves at source-drain bias voltages of 0.5 V and −0.5 V. [b]The molecular resistances are obtained from the whole resistances of the rejoined devices subtracted by those of the pristine graphene devices before cutting.

Supplementary Table 4 | Summary of the resistance values of rejoined junctions based on the control terphenyl molecule.

Device number	Current (nA)[a]	Molecular resistance (MΩ)[b]	Molecular conductance ($10^{-4} \times G_0$)
1	6.85	72.99	1.77
2	43.39	11.52	11.20
3	81.69	6.12	21.08
4	80.61	6.20	20.81
5	35.06	14.26	9.05
6	61.09	8.18	15.77
7	68.00	7.35	17.55

[a]The current values are obtained by averaging the data from *I-V* curves at source-drain bias voltages of 0.5 V and −0.5 V. [b]The molecular resistances are obtained from the whole resistances of the rejoined devices subtracted by those of the pristine graphene devices before cutting.

4. Supplementary References

[1] Jia, C. *et al.* Switching and mechanisms in single-molecule junctions. *Angew. Chem. Int. Ed.* **52**, 8666–8670 (2013).

[2] Wen, H. *et al.* Complex formation dynamics in a single-molecule electronic device. *Sci. Adv.* **2**, e1601113 (2016).

[3] Xin, N. *et al.* Thermally activated tunneling transition in a photoswitchable single-molecule electrical junction. *J. Phys. Chem. Lett.* **8**, 2849–2854 (2017).
